# Processing and release of endogenous immunogenic peptide signals

**DOI:** 10.1111/tpj.70897

**Published:** 2026-05-10

**Authors:** Maurice Koenig, Hermanyanto Laia, Gunther Doehlemann, Johana C. Misas Villamil

**Affiliations:** ^1^ Institute for Plant Sciences and Cluster of Excellence on Plant Sciences (CEPLAS) University of Cologne Cologne Germany

**Keywords:** phytocytokines, cytokines, signaling, cell communication, proteases

## Abstract

In both animals and plants, small secreted peptides known as cytokines and phytocytokines mediate local and systemic communication during immune and stress responses. These signaling molecules are typically synthesized as inactive precursors that require proteolytic processing to become active. They are subsequently perceived by membrane‐localized receptors that coordinate defense and developmental signaling pathways. While the maturation and function of cytokines are well understood in animals, the mechanistic understanding of phytocytokine processing and release in plants is limited. Predicting cleavage sites or identifying the responsible proteases remains challenging, and how phytocytokines move as long‐distance or systemic signals is a relatively new area of exploration. In here, we discuss the parallels between cytokines and phytocytokines, including their properties as small, secreted peptides activated by proteolysis and perceived by specific receptors to modulate immunity. Finally, we address the divergence and evolution of endogenous peptide signals, emphasizing the molecular arms race between hosts and microbes. Together, these evolutionary dynamics and the functional similarities between cytokines and phytocytokines reveal striking examples of convergence and highlight important gaps in our understanding of how plants process, release, transport, and perceive these immune peptides.

## Significance Statement

Plant endogenous peptides, termed phytocytokines, coordinate immune responses and mediate intercellular communication within and across plant tissues. While an expanding repertoire of ligands, receptors, and signaling pathways has been identified, key aspects of phytocytokine biogenesis and regulation remain unresolved. By comparing plant peptide signals with animal cytokines, this review highlights shared principles and fundamental differences and identifies proteolytic processing, peptide release, and spatiotemporal signaling as critical but understudied determinants of immune specificity in plants.

## (PHYTO)CYTOKINES ARE IMMUNE‐RELATED PEPTIDES IN PLANTS AND ANIMALS

Throughout evolution, the biology of hosts and pathogens has been shaped by each other, resulting in the emergence of complex immune signaling systems that must respond rapidly yet remain tightly controlled. Microbe‐associated molecular patterns (MAMPs), including bacterial components such as flagellin (flg22), elongation factors (EF‐Tu), peptidoglycan (PG), and lipopolysaccharides (LPS), as well as fungal‐derived molecules such as chitin and β‐glucans, are conserved molecular signatures within microbial taxa that are recognized by pattern recognition receptors (PRRs) to trigger immune responses (Newman et al., 2013). Host recognition of MAMPs via PRRs is crucial for immune activation. Pathogens can cause damage during penetration, triggering the production of damage‐associated molecular patterns (DAMPs) from the host. DAMPs comprise endogenous molecules that alert the immune system to cellular damage. In plants, these include passively released signals, such as oligogalacturonides (OGs), extracellular ATP, and NAD(P), and actively released signaling peptides termed phytocytokines (Ge et al., 2022; Pastor et al., 2022; Roudaire et al., 2021). Phytocytokines are often proteolytically released from precursor molecules into the apoplast when plants experience stress such as pathogen attack or damage (Hou, Liu, & He, 2021). In the apoplast, the released peptides serve as signaling components in immunity, similar to animal cytokines. Accordingly, phytocytokines execute cytokine‐like roles in plants, paralleling animal cytokines that mediate immune signaling and intercellular communication (Luo, 2012; Murtaugh et al., 1996). In this manuscript we define phytocytokines as endogenous immunogenic peptide signals proteolytically released from precursor proteins shown to be involved in plant immunity.

We highlight five common characteristics of cytokines and phytocytokines: First, they are typically small peptides or proteins eliciting a biological response after recognition. Second, they are often synthesized as inactive precursors that require proteolytic cleavage for activation. Third, they are released into the extracellular space and function in cell‐to‐cell communication, acting on target cells in an autocrine (cell‐autonomous), paracrine (local tissue), or endocrine (systemic tissue) manner. Fourth, they serve as extracellular signaling molecules, recognized by specific receptors that initiate downstream immune responses. Finally, they act as immune modulators, coordinating defense pathways and fine‐tuning immune responses. Moreover, they can even be hijacked by microbes for their own benefit. In the following sections, these features are discussed in greater detail.

## (PHYTO‐)CYTOKINES ARE SMALL PROTEINACEOUS MOLECULES

Cytokines are typically small signaling proteins or peptides (5–25 kDa) that encompass diverse structural families, such as four‐helix bundles, β‐sheets, and cysteine knots. These families include chemokines, interferons, interleukins (IL), tumor necrosis factors (TNF), colony‐stimulating factors, lymphokines, and monokines (Kaiser & Stäheli, 2014; Liu et al., 2021; Stenken & Poschenrieder, 2015). Similarly to cytokines, most phytocytokines are small peptides, some of which consist of just a few amino acids. Examples of these are the Arabidopsis PSK1 (Phytosulfokine 1), which consists of five amino acids (YIYTQ), and the largest group to date, the RALF (Rapid Alkalinization Factor) peptides, which contain up to 50 amino acids (~6 kDa).

The small, proteinaceous nature of endogenous signaling peptides has important functional implications for their mobility and signaling range. Their limited size allows them to diffuse and mediate local (paracrine) and systemic (endocrine) signaling. Local diffusion has been shown for Arabidopsis Pep1 (Plant Elicitor Peptide 1, 23 amino acids) which diffuses in leaves, likely also forming a gradient in distal tissues, to trigger defense responses in a similar manner as chemokines (8–12 kDa) do (Huffaker et al., 2006; Zlotnik & Yoshie, 2012). Systemic diffusion has been shown for Systemin (18 amino acids) which is transported via the phloem and activates jasmonate‐dependent responses in distal leaves (Pearce et al., 1991). Moreover, peptide synthesis and turnover often is tightly regulated, enabling dynamic control of signaling outputs. For example, Peps are rapidly activated upon cell damage, and their signaling is transient, suggesting efficient attenuation mechanisms, although their extracellular stability and degradation kinetics remain poorly defined (Baggiolini et al., 1989; Hander et al., 2019). Finally, their short peptide sequences contain distinct interaction motifs conferring high receptor‐binding specificity. This is exemplified by the requirement of the “SxS” motif of the SERINE RICH ENDOGENOUS PEPTIDES (SCOOPs) to bind to the leucine‐rich repeat receptor kinase MALE DISCOVERER 1‐INTERACTING RECEPTOR‐LIKE KINASE 2 (MIK2) in the model plant *Arabidopsis thaliana* (Snoeck et al., 2024).

The small size and tight regulation of phytocytokines make their detection, quantification, and characterization technically challenging. Mass spectrometry is widely used to identify proteins and diverse endogenous or pathogen‐derived peptides. Immunoassays such as ELISA have been commonly used in clinical diagnostics and animal research to detect and quantify cytokines (Chiswick et al., 2011), but for plants, the establishment of ELISA methods has been challenging. Recently, the use of a Zip1‐specific antibody to monitor Prozip1 processing and Zip1 release has been established (Koenig, Sorger, Keh, et al., 2025).

## PHYTOCYTOKINES REQUIRE PROTEOLYTIC ACTIVATION

A key similarity between cytokine signaling in animals and phytocytokine signaling in plants is their proteolytic activation, which allows the controlled release of mature signaling peptides. The cytokines of the interleukin‐1 family (IL‐1) and tumor necrosis factor (TNF) are synthesized as precursor proteins that require proteolytic cleavage to gain receptor‐binding competence and initiate downstream signaling (Dinarello & Margolis, 1995). Many IL‐1 family members are produced as inactive precursors (pro‐cytokines) containing N‐terminal pro‐domains that prevent receptor engagement until proteolytic processing occurs. A classical cytokine activation example is IL‐1β, which is produced as a 31‐kDa precursor and requires processing by caspase‐1 (cysteine aspartate‐specific protease) to release the active 17 kDa bioactive form. In addition to this canonical caspase‐1–dependent pathway, alternative proteolytic routes can contribute to pro‐IL‐1β processing, involving caspase‐3, caspase‐7, and caspase‐8, as well as extracellular or granule‐associated serine proteases such as chymase, tryptase, cathepsin G, proteinase 3, neutrophil elastase, and granzyme B (Bauernfried et al., 2025; Gurung & Kanneganti, 2015; Keitelman et al., 2022; Netea et al., 2010). Caspase cleavage typically occurs after an aspartic acid (Asp, D) residue. The canonical motif most commonly recognized by the inflammatory caspase‐1 includes the tetrapeptide sequence WEHD or YVAD (Rotonda et al., 1996; Wilson et al., 1994), whereas the executioner caspase‐3 recognizes the motif DEVD (Thornberry et al., 1997). This substrate selectivity arises from differences in the S4 pocket of their catalytic sites (Julien & Wells, 2017). The proteolytic activation of cytokines is often a regulatory mechanism preventing premature or inappropriate activation of potent pro‐inflammatory signals (Afonina et al., 2015; Zheng et al., 2020; Zhu & Kanneganti, 2017).

**Figure 1:**
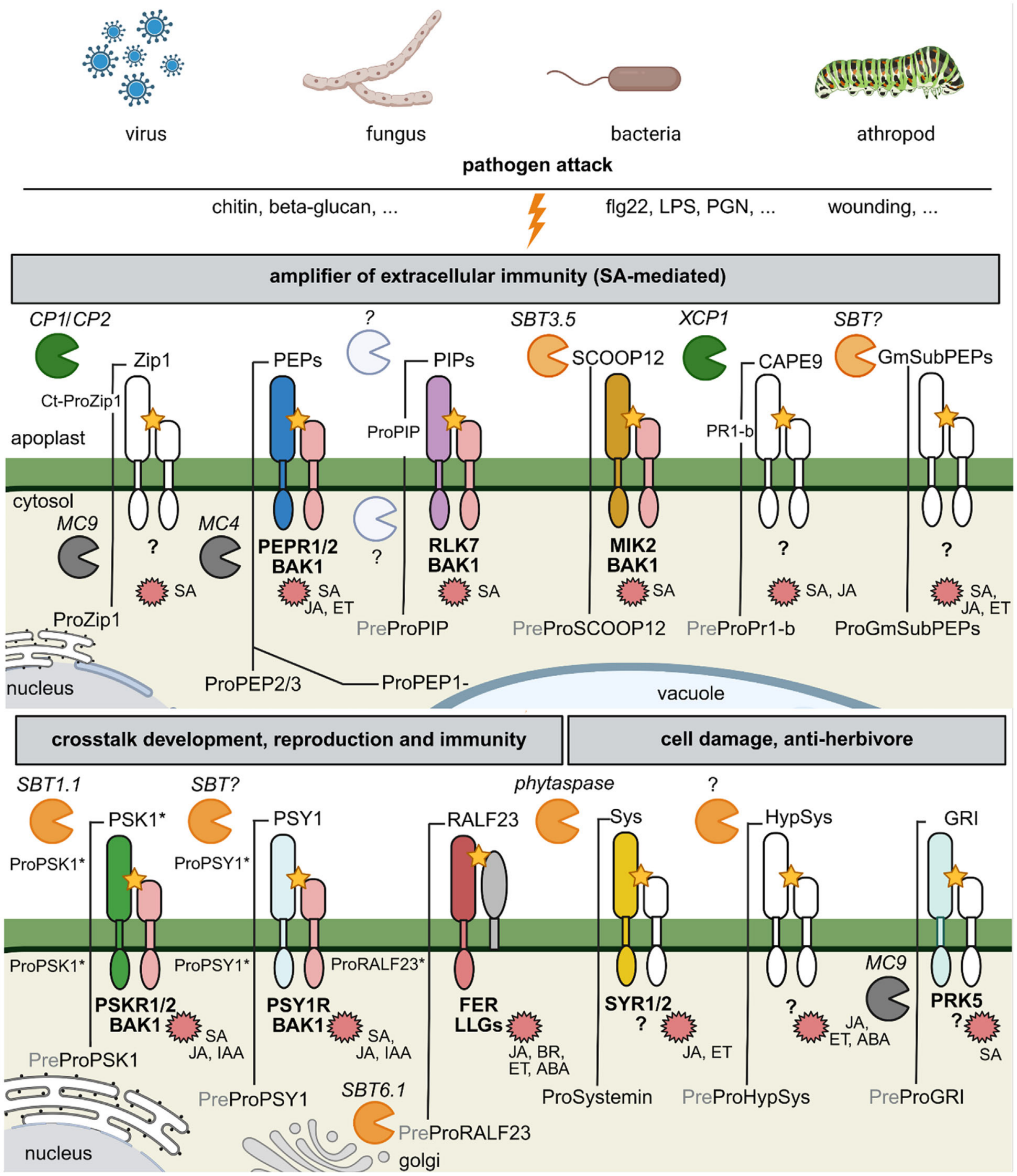
Examples of phytocytokines, their corresponding (co‐)receptors, processing proteases and phytohormonal responses. After pathogen attack or damage phytocytokines can serve as amplifiers of extracellular immunity, others allow the crosstalk between development and immunity, regulate growth or signal in response to wounding. Papain‐like cysteine proteases (PLCPs) (green), subtilases (SBTs) (orange), metacaspases (MCAs) (gray). Stars indicate the perception of the mature bioactive phytocytokine. Red starbursts indicate the hormonal pathway activated after perception. Figure was created with BioRender.

Unlike metazoans, which encode caspases that mediate cytokine cleavage and other key apoptotic and inflammatory processes, plants lack caspases but possess several enzymes with caspase‐like activity. The proteolytic processing of phytocytokine precursors is essential for the release of bioactive peptides because the majority of identified phytocytokines are derived from precursor proteins without an additional known cellular function. So far, three protease families have been clearly implicated in phytocytokine maturation: (i) papain‐like cysteine proteases, (ii) metacaspases, and (iii) subtilases (Figure 1, Table 1). Several reviews comprehensively address plant protease activation, regulation, and localization, highlighting that plant proteases are synthesized as inactive precursors and become activated in a spatially and temporally controlled manner through specific cues and post‐translational modifications. Collectively, they describe diverse protease families and outline emerging mechanistic principles governing protease activation in immunity and development (examples in Fernández‐Fernández et al., 2023; van der Hoorn, 2008; van der Hoorn & Klemenčič, 2021). Below, we will describe the role of the three main protease families in phytocytokine activation.

**Table 1:** Known cleavage sites of plant phytocytokines

Peptide	Species	Cleavage sites (P_4_P_3_P_2_P_1_↓P_1_′P_2_′P_3_′P_4_′)	Protease	First peptide identification	References
CAPE9	Arabidopsis	CNYD↓**PRGN**	XCP1	Bioinformatics	Chen et al. (2023)
Wip1	Wheat	ITRR↓**SPLD…PKPG**↓RRPD	RD21A	MS analysis	Liu et al. (2023)
Zip1	Maize	FR↓(extracellular)^b^	CP1 and CP2	MS analysis	Ziemann et al. (2018)
		RR↓ (intracellular)^b^	ZmMC9		Koenig, Sorger, Mantz, et al. (2025)
GRIp	Arabidopsis	SKTR↓**LLVS…KIKK**↓GMRC^a^	AtMC9	Bioinformatics	Wrzaczek et al. (2015)
Pep1	Arabidopsis	VTSR↓**ATKV**	AtMC4	Bioassay (alkalinisation activity) ‐ MS	Huffaker et al. (2006) and Hander et al. (2019)
Pep3 Pep4	Arabidopsis	GKNK↓**TKPT**	AtMC4	Bioinformatics	Shen et al. (2019), Nakaminami et al. (2018) and Hander et al. (2019)
RALF23	Arabidopsis	RRIL↓**ATRR…NTIP**↓CSRR	SBT6.1	Bioinformatics	Olsen et al. (2002), Srivastava et al. (2009) and Ghorbani et al. (2016)
PSK1	Arabidopsis	AHTD↓**YIYTQ**↓DLNL	SBT3.8 SBT1.1	MS analysis	Matsubayashi and Sakagami (1996) and Stührwohldt et al. (2021)
PSK4	Arabidopsis	SLVL↓**HTDY**	SBT3.8 SBT1.1	Bioinformatics	Srivastava et al. (2008)
CLE40	Arabidopsis	EVEE↓**RQVT…PLHH(K)**↓HIPF	SBT1.4, SBT1.7, SBT4.13	Bioinformatics	Stahl et al. (2009), Stührwohldt et al. (2020a) and Stührwohldt et al. (2020b)
GLV1/CLEL6	Arabidopsis	RRAL↓GGVE**…DYPQ…**	SBT6.1	Bioinformatics	Whitford et al. (2012), Ghorbani et al. (2016), Stührwohldt et al. (2020a) and Stührwohldt et al. (2020b)
IDA	Arabidopsis	YLPK↓**GVPI…SRHN**↓SFVN	SBT4.12, SBT4.13, SBT5.2	Bioinformatics	Stenvik et al. (2008) and Schardon et al. (2016)
Systemin	Tomato	REDL↓**AVQS…MQTD**↓NNKL	Phytaspases	SCX HPLC—Amino Acid Analysis	Pearce et al. (1991) and Beloshistov et al. (2018)
SCOOP12	Arabidopsis	RRLM↓GSGA**…PVRS** ^b^	SBT3.5	Bioinformatics	Gully et al. (2019) and Yang et al. (2023)

Amino acids in bold are the (start of) mature peptide. Colors indicate the class of protease: Papain‐like cysteine proteases (PLCPs, green), metacaspases (MCAs, gray), subtilases (SBTs, orange).

^a^ Other cleavage sites have also been also identified.

^b^ Not precisely mapped.

### Papain‐like cysteine proteases (PLCPs)

PLCPs are members of clan CA, family C1 of cysteine proteases with papain from *Carica papaya* as its type peptidase (Rawlings et al., 2018). This family comprises predominantly endopeptidases, and the catalytic activity relies on a conserved Cys‐His–Asn triad. Substrate specificity is largely determined by the S2 pocket, which typically favors bulky hydrophobic residues and disfavors charged side chains (Choe et al., 2006; Rawlings et al., 2018). Plant PLCPs usually contain a signal peptide for secretion and are synthesized as inactive pre‐pro‐enzymes (zymogens), becoming active predominantly in the apoplast, cell wall‐associated compartments, or vacuoles (Richau et al., 2012). A well‐characterized example of a PLCP releasing a phytocytokine is the Xylem Cysteine Peptidase 1 (XCP1) from Arabidopsis, which cleaves PR1 (pathogenesis‐related 1) at the CYND‐motif to release the CAPE9 peptide (Chen et al., 2023). Interestingly, the processing of CAPE peptides in other plant species is not yet clear. For example, the maize XCP2 protease, an ortholog of AtXCP1, does not cleave the PR1‐like precursor PRB1‐3 to release ZmCAPE (Lin et al., 2023). Instead, the maize pathogen *Ustilago maydis* hijacks the maize PLCP CathB to cleave UmPR‐1La at a similar CYNX‐motif, releasing a UmCAPE‐like peptide (Lin et al., 2023). Protease specificity does not only rely on the recognition of a certain motif, as shown in the maize—*U. maydis* interaction, as the maize PRB1‐3 contains the same CYNX‐motif, but it is not cleaved by ZmCathB (Lin et al., 2023). Indeed, *Nicotiana benthamiana* CathB acts mainly as an endopeptidase different than its animal counterpart, which mostly cleaves C‐terminal dipeptides (Niemer et al., 2016). In wheat, *Triticum aestivum*, TaRD21 has been identified as the protease releasing Wip1 from its precursor ProWip1, enhancing wheat resistance to wheat yellow mosaic virus. RR/FR motifs were identified as cleavage sites of TaRD21, and Wip1 release depends on processing at these motifs (Liu et al., 2023). In maize, double arginine motifs were implicated in Prozip1 processing, and the PLCPs CP1 and CP2 were initially proposed to mediate Zip1 release, thereby reinforcing SA accumulation through a positive feedback loop (Ziemann et al., 2018). Later studies showed that Prozip1 processing is initiated intracellularly and requires metacaspase activation for Zip1 release (Koenig, Sorger, Mantz, et al., 2025).

### Metacaspases (MCAs)

MCAs are cysteine proteases of clan CD, which also comprises caspases and paracaspases. Their active site is defined by a conserved His–Cys catalytic dyad, and, like all clan CD proteases, they exhibit a strict requirement for the residue in the P1 position. In contrast to caspases, which cleave substrates after aspartate (D) residues, MCAs (family C14B) preferentially cleave after basic residues such as arginine (R) or lysine (K) (Rawlings et al., 2018). Plant MCAs generally lack signal peptides and localize to the cytosol or other intracellular compartments, raising the possibility that phytocytokine precursors targeted by MCAs are processed inside the cell prior to extracellular release. MCAs are synthesized as zymogens and activated by proteolytic cleavage into p20 and p10 subunits, which assemble into an active enzymatic complex; in many cases, their activity is further regulated by calcium cations (Zhu et al., 2020). Type II MCAs, have been shown to release phytocytokines from their precursor molecules. One example is the Arabidopsis metacaspase 4 (AtMC4/AtMCA‐IIa) which processes Arabidopsis Propep1 at arginine (R^69^), releasing Pep1 (Flury et al., 2013; Hander et al., 2019; Huffaker et al., 2006; Shen et al., 2019; Yamaguchi et al., 2010). Arabidopsis Pep1 (AtPep1) is a prototypical phytocytokine that amplifies pattern‐triggered immunity, mediates damage signaling, primes systemic defenses, and integrates hormone responses, through perception by the leucine‐rich repeat receptor kinases PEPR1 and PEPR2 (Figure 1) (Bartels et al., 2013; Huffaker et al., 2006; Yamaguchi et al., 2010). Recently, it has been demonstrated that Peps also play a role in wheat (*T. aestivum*) immunity, and that their processing, release and perception mechanism are similar to those of AtPep1. Wheat Peps (TaPeps) are processed at a conserved Arg residue by TaMCA‐IIa, a type II MCA ortholog of AtMC4, releasing a bioactive phytocytokine that is perceived by the TaPEPR1 receptor contributing to wheat immunity and resistance against *Fusarium* head blight (FHB) (Dong et al., 2025). While direct biochemical evidence is not yet available, it is plausible that maize Peps are processed by type‐II MCAs, similar to what has been demonstrated in Arabidopsis and in wheat. This points toward an evolutionarily conserved mechanism of Pep maturation in both dicots and monocots. In *Solanaceae*, the wound‐induced peptide REF1, a PROPEP‐family SlPep, acts as a primary local wound signal that promotes tissue regeneration at wound sites (Yang et al., 2024), however, the mechanism of REF1 precursor processing into the mature peptide has not been demonstrated.

In contrast to the widely conserved Peps, GRIM REAPER peptides (GRIp) are found only in a limited number of plant species, predominantly within the *Brassicaceae*, suggesting that GRIp may represent a lineage‐specific branch of the phytocytokine repertoire. The GRIM REAPER precursor protein (GRI) belongs to the STIG (stigma‐specific) family of small secreted proteins, which have been implicated in reproductive and developmental processes, including pollen‐associated signaling and embryo surface patterning cuticle (Wrzaczek et al., 2015; Zhang et al., 2017). In Arabidopsis, GRI is proteolytically processed by the metacaspase AtMC9 (AtMCA‐IIf) at positions R^67^ and K^78^ to release bioactive GRIp, which induces reactive oxygen species (ROS), activates salicylic acid (SA) signaling, and promotes programmed cell death via the receptor‐like kinase PRK5 (Wrzaczek et al., 2009, 2015).

Recently, another lineage‐specific phytocytokine, Prozip1, has been shown to be intracellularly processed through arginine‐specific cleavage by the maize type II calcium‐dependent metacaspase ZmMC9 (Koenig, Sorger, Mantz, et al., 2025). Zip1 release relies on a two‐step mechanism in which metacaspase‐dependent N‐terminal processing occurs intracellularly, thereby enabling the translocation of the C‐terminal fragment (Ct‐Prozip1) into the apoplast. In the apoplast, Ct‐Prozip1 could be further processed by apoplastic PLCPs, which could release and degrade the Zip1 peptide (Koenig, Sorger, Mantz, et al., 2025).

### Subtilases (SBTs)

SBTs are serine proteases belonging to the clan SB and family S8A, with subtilisin from *Bacillus licheniformis* serving as the type member. The family S8, referred to as subtilases, represents the second largest family of serine peptidases, with regard to both the number of sequences and the number of characterized peptidases. The S8 family is divided into two subfamilies. Subtilisin is the type‐example for subfamily S8A, and kexin is the type‐example for subfamily S8B (Rawlings et al., 2018). SBTs have been found to contain a catalytic triad comprising the amino acids in the order Asp–His–Ser. The majority of family members are classified as endopeptidases, exhibiting optimal activity at neutral to mild alkali pH and a pronounced affinity for cleaving after hydrophobic residues (Rawlings et al., 2018). Plant SBTs are divided in six subgroups, SBT subfamilies 1–6, based on phylogenetic analysis and gene structure with the exception of phytaspases which make a unique clade (Schaller et al., 2018). Most plant SBTs possess a signal peptide although their activation can happen in different cellular compartments, such as the trans‐Golgi network or the apoplast (Schaller et al., 2018). Apoplastic alkalinization triggered by pathogen perception or tissue damage creates conditions that favor subtilase activation and processing of peptide precursors (Gust et al., 2017; León et al., 2001; Newman et al., 2013; Zhou et al., 2020). Notably, phytaspases can relocalize from the apoplast to intracellular compartments upon stress, where they contribute to intracellular proteolytic cascades associated with programmed cell death (Chichkova et al., 2010; Schaller et al., 2018; Vartapetian et al., 2011).

Processing of Systemin, the first signaling peptide found in plants, is mediated by phytaspases, aspartate‐specific proteases with a caspase 6‐like cleavage specificity (Beloshistov et al., 2018). Systemin is released from its precursor protein Prosystemin, that lacks an N‐terminal secretion signal, by wounding or insect attack (McGurl et al., 1994). Tomato phytaspase exhibits a remarkable specificity by recognizing extended D‐containing amino acid motifs, rather than individual D residues in its targets. The identification of preferentially hydrolyzed VNLD‐ and VEID‐based substrates served as basis for the characterization of the motifs VRED and MQTD flanking the released of the 18 amino acids bioactive peptide Systemin (Beloshistov et al., 2018). Subtilases are also involved in the release of RALFs, peptides involved in cell wall integrity and immune regulation (Figueiredo et al., 2018; Srivastava et al., 2009). In Arabidopsis, RALF23 is released from its precursor protein within the Golgi prior to secretion by AtSBT6.1, which recognizes the motif RRIL (Srivastava et al., 2008; Stegmann et al., 2017). The same protease and its homolog AtSBT6.2 have been identified to release the GOLVEN peptide GLV1 (also referred to as RGF/CLEL family peptide), a secreted peptide class initially defined through the discovery of tyrosine‐sulfated RGFs and subsequent characterization of GLV and CLEL peptides (Matsuzaki et al., 2010; Meng et al., 2012; Whitford et al., 2012). GLV1 mediates cell elongation and promotes cell surface immune receptor abundance after recognition by the RGI receptor (Ghorbani et al., 2016; Stegmann et al., 2022). AtSBT6.1 and AtSBT6.2 are orthologs of mammalian site‐1 protease (S1P), which plays a role in the unfolded protein response and endoplasmic reticulum (ER) stress. They are also orthologs of tripeptidyl peptidase II (TPP2), which plays a role in antigen processing (Schaller et al., 2018). Different families of subtilases, AtSBT1.1, AtSBT3.8 and the tomato phytaspase SlPhyt2 have been shown to release the disulfate pentapeptide AtPSK4, AtPSK1, and tomato PSK, respectively (Reichardt et al., 2020; Srivastava et al., 2008; Stührwohldt et al., 2021). PSKs have been associated with growth, development and immunity (Igarashi et al., 2012; Li et al., 2024). PSK signaling has recently been shown to restrain SA‐dependent immune activation during beneficial rhizosphere colonization, thereby contributing to growth‐defense trade‐off control (Song et al., 2023). In Arabidopsis, the subtilase cleavage of PSKs appeared to be protease‐specific as the apoplastic AtSBT1.1 exclusively cleaves AtPSK4 leaving three residues at its N‐terminus (Table 1), but no other AtPSKs (Srivastava et al., 2008), similarly AtSBT3.8 is highly specific to AtPSK1. This specificity results from the presence of an aspartic acid residue directly following the PSK1 peptide sequence (Stührwohldt et al., 2021; Table 1). In contrast, the release of all seven PSK peptides in tomato depends on the phytaspase SlPhyt2 Asp‐specific cleavage (Reichardt et al., 2020).

The STB3 subtilase subfamily is implicated in releasing CLV3/ESR‐RELATED (CLAVATA3 (CLV3)/EMBRYO SURROUNDING REGION‐RELATED) (CLE) peptides, one of the largest plant signaling peptide families involved in development and immunity. The *A. thaliana* genome contains 32 CLE genes (Nakagami et al., 2024). CLE precursors include an N‐terminal signal peptide and a conserved 14‐aa C‐terminal CLE motif. Positional residue analyses show that an invariant P1′ arginine and acidic residues at P2/P3 are essential for efficient cleavage (Ni & Clark, 2006). Inhibitor studies indicate that subtilases mediate N‐terminal processing, while carboxypeptidases contribute to C‐terminal trimming (Ni et al., 2011). Subtilases also process CLE‐like (CLEL) peptides, with SBT6.1 and SBT3.8 performing sequential maturation steps along the secretory pathway to generate the final 12–14 aa active peptides (Stührwohldt et al., 2020a, 2020b). The release of SCOOPs also requires SBT3‐mediated cleavage. SCOOP peptides are involved in the regulation of stress responses, root growth promotion, and immune defense activation (Gully et al., 2019; Rhodes et al., 2021; Stahl et al., 2022; Wang, Chen, et al., 2024; Wu et al., 2024). Subtilase‐mediated cleavage occurs at conserved N‐terminal motifs within Proscoop precursors, and disruption of these subtilases or their recognition sequences abolishes Proscoop processing and peptide maturation (Wu et al., 2024; Yang et al., 2023). Most Proscoop precursors contain an RxLx or RxxL motif that has been proposed to be specifically recognized and cleaved by subtilases. For example, SBT3.5 cleaves Proscoop12 at the RRLM motif (del Corpo et al., 2024; Yang et al., 2023). Proscoop20 lacks this motif but features a conserved VWD motif cleaved by the subtilases SBT3.6, SBT3.8, and SBT3.9.

Three members of the SBT4 and SBT5 subfamilies (AtSBT5.2, AtSBT4.12, AtSBT4.13) have been implicated in cleaving the N‐terminus of the mature IDA (Inflorescence Deficient in Abscission) peptide mostly implicated in the abscission of sepals, petals, and stamens in Arabidopsis flowers (Schardon et al., 2016; Stührwohldt et al., 2018) but with a dual function in immunity likely to guard the cells undergoing separation from pathogen attack (Lalun et al., 2024). Using Ala‐substituted P1–P4 positions of an elongated IDA peptide (eIDA) as analog substrates of SBT4.13, the positions P2‐Pro and P4‐Tyr were confirmed as the two most important residues for the formation of the mature IDA peptide (mIDA) (Schardon et al., 2016). The proteases cleaving the C‐terminal of the mIDA precursor still remain to be elucidated, although a carboxypeptidase has been proposed (Tamaki et al., 2013). Interestingly, the C‐terminal Asn residue is conserved in several other peptide families including the CLE, GLV, and PEP families probably due to its requirement for receptor binding (Zhang et al., 2016).

In most cases, attributing the release of a bioactive peptide to a single protease is challenging. This complexity is particularly evident among subtilases (SBTs), where multiple family members contribute redundantly to the maturation of phytocytokines such as GLV, PSK, CLE, and IDA (Table 1).

### What are the determinants of cleavage specificity?

Although proteolytic cleavage is essential for phytocytokine activation, precise mature forms have been directly identified by mass spectrometry for only a limited set of peptides (Table 1). For many phytocytokines, the exact processing events *in planta* remain incompletely defined, underscoring the need to understand the determinants of cleavage specificity. Several proteases and their corresponding cleavage sites have been described for different phytocytokines, but the sequence specificity that governs phytocytokine maturation remains unclear (Figure 1; Table 1). Cleavage motifs often overlap between different protease classes, which makes it difficult to assign a given site to a specific enzyme. This limited sequence determinism is consistent with proteomics studies showing that many apoplastic proteases display broad substrate tolerance and lack strict sequence‐specific recognition motifs (Kaschani et al., 2010; Misas‐Villamil & van der Hoorn, 2008; Sueldo et al., 2024). As a consequence, it remains difficult to predict which protease will process a particular precursor to release its bioactive peptide. Recent structural and biochemical studies suggest that cleavage of phytocytokine precursors commonly takes place in locally flexible or intrinsically disordered regions, where sequence motifs and structural accessibility act together to define protease specificity. For example, the pathogenesis‐related protein PR1b, adopts a compact β‐barrel structure, but its C‐terminal tail harboring the CAPE motif is intrinsically disordered and accessible to PLCPs (Breen et al., 2017; Chen et al., 2014, 2023). In contrast, the two metacaspase substrates Propep1 and Prozip1 (Koenig, Sorger, Mantz, et al., 2025) are predicted to be largely unstructured. This is consistent with the tendency of MCAs to cleave accessible sites within flexible or disordered regions (Hander et al., 2019; Zhu et al., 2020). Subtilases might act in intermediate contexts, cleaving short, semi‐flexible linkers between structured domains, as shown for Proralf23 (Srivastava et al., 2009; Stegmann et al., 2017). These findings indicate that local structural dynamics, together with conserved sequence motifs, might be the primary determinants of phytocytokine processing and release. Mass spectrometry‐based peptidomics shows that some phytocytokine precursors can generate multiple peptide forms rather than a single mature product (Patel et al., 2018; Zhang et al., 2025). Although this has been demonstrated for only a few families, it suggests that the exact processing outcome may depend on the cellular or environmental context, such as tissue type, developmental stage, or stress conditions. Together, this raises the key question of how specific cleavage events are selected *in vivo*, and which molecular determinants, such as precursor sequence, protease specificity, subcellular localization, and local physicochemical conditions, bias processing toward bioactive peptide forms.

Proteolysis control and release of bioactive molecules can also be modulated by post‐translation modifications (PTMs). Many cytokine PTMs regulate their stability, activity, secretion, and receptor interactions. Common PTMs observed in cytokines include glycosylation, phosphorylation, acetylation, and ubiquitination among others (Zhong et al., 2023). Cytokines such as IL‐2, IL‐6, IL‐7, IL‐10, and TNF‐α undergo N‐linked or O‐linked glycosylation. Glycosylation affects their folding, stability, and receptor binding (Afonina et al., 2022). One prominent example is the ubiquitination of IL‐1β that limits cleavage by caspase‐1 and promotes its proteasomal degradation (Vijayaraj et al., 2021). Although in plants there are not yet clear examples of PTMs that abolish phytocytokine release, it has been shown that PTMs can promote peptide maturation. For example, processing of the plant peptide hormone TWS1 (Twisted seed 1) by subtilases requires prior tyrosine sulfonation for efficient protease recognition and correct processing (Royek et al., 2022). Besides, PTMs can be relevant for peptide activity, as they affect peptide secretion, peptide stability, or peptide–receptor interactions (Stührwohldt et al., 2018). For example, specific N‐glycans on MIK2 directly interact with the co‐receptor BRASSINOSTEROID‐INSENSITIVE 1‐ASSOCIATED RECEPTOR KINASE 1 BAK1 upon SCOOP sensing thereby enabling SCOOP‐triggered immune signaling (Wu et al., 2024). Known phytocytokines that require PTMs are PSKs, PSYs, CLEL peptides, and PIPs, which undergo sulfonation, hydroxylation, glycosylation and arabinosylation of the hydroxyproline residue (Matsubayashi, 2014; Stührwohldt & Schaller, 2019b) enhancing their bioactivity via receptor interaction. Arabinosylation of hydroxyproline (Hyp) is an important post‐translational modification found on secreted CLEL peptides and this PTM is critical for their function (Nakagami et al., 2024). Thus, PTMs represent an additional regulatory layer controlling peptide bioavailability and receptor engagement, although their relevance for many phytocytokines is still poorly understood.

## MECHANISMS OF PEPTIDE EXTRACELLULAR RELEASE AND SECRETION

Cytokine secretion typically occurs via the classical ER‐Golgi pathway. Canonical cytokines with N‐terminal signal peptides, such as TNF‐α and IL‐6, are folded in the ER, processed in the Golgi, and trafficked to the plasma membrane through clathrin‐coated carriers, the trans‐Golgi network, and VAMP3‐positive recycling endosomes. This pathway is stimulus‐dependent and can be disrupted by inhibitors of ER‐to‐Golgi transport such as Brefeldin A (Kaminska et al., 2024). Non‐classical secretion pathways predominate for ‘leaderless’ cytokines, such as IL‐1β and IL‐18, as their precursors lack signal peptides and bypass the ER‐Golgi system. These cytokines are released through gasdermin D‐formed membrane pores, within extracellular vesicles, or during pyroptotic cell rupture (Nie et al., 2025). Similarly, neutrophils release cytosolic cytokines such as MIF and IL‐16 upon necrosis (Roth et al., 2016; Tecchio et al., 2014). Comparable to cytokines, phytocytokines can be classified based on the presence of an N‐terminal signal peptide in their precursors (Gust et al., 2017; Matsubayashi, 2014). For example, precursors of HypSys, PIPs, SCOOP12, PSKs, PSY1, CAP‐derived peptides (CAPEs), and RALFs contain a predicted signal peptide and enter the secretory pathway, making them canonical secreted precursors. In contrast, precursors of Systemins, PEPs, soybean GmSubPeps, and Zip1 lack a predicted signal peptide and are considered non‐canonical secreted precursors (Figure 1). Phytocytokine precursors carrying N‐terminal signal peptides are routed into the ER‐Golgi secretory pathway, while many non‐canonical precursors can associate with organelle membranes or microsomal fractions. For example, Arabidopsis Propep1 and Propep6 reside on the tonoplast, the cytosolic face of the vacuolar membrane (Bartels et al., 2013).

The mechanisms by which phytocytokines with non‐canonically secreted precursors reach the extracellular space are poorly understood. Two non‐exclusive scenarios are plausible: passive release from damaged cells followed by proteolysis as proposed for Peps (Hander et al., 2019), or active export via non‐classical secretion. In the non‐classical secretion pathways, phytocytokines could traffic to the apoplast via extracellular vesicle‐mediated transport regulated by Rab GTPases (Lu et al., 2024; Shi et al., 2023).

Most non‐canonically secreted phytocytokine precursors lack conserved structural domains and appear to act primarily as carriers for their bioactive peptide (Hou, Liu, & He, 2021). However, some precursors may exert additional roles in plant defense. In the case of Prosystemin, proteolytic fragments derived from the precursor activate immune responses, and Prosystemin‐derived peptides (PDPs) can induce defense gene expression independently of Systemin itself (Beloshistov et al., 2018; Molisso et al., 2022). Conversely, certain secreted precursors such as PR‐1 possess intrinsic antimicrobial activity through sterol binding (Gamir et al., 2017; Han & Schneiter, 2024), highlighting that some precursor proteins may hold functions independent from phytocytokine release.

## PHYTOCYTOKINE PERCEPTION, DIVERSITY AND EVOLUTION

**Figure 2:**
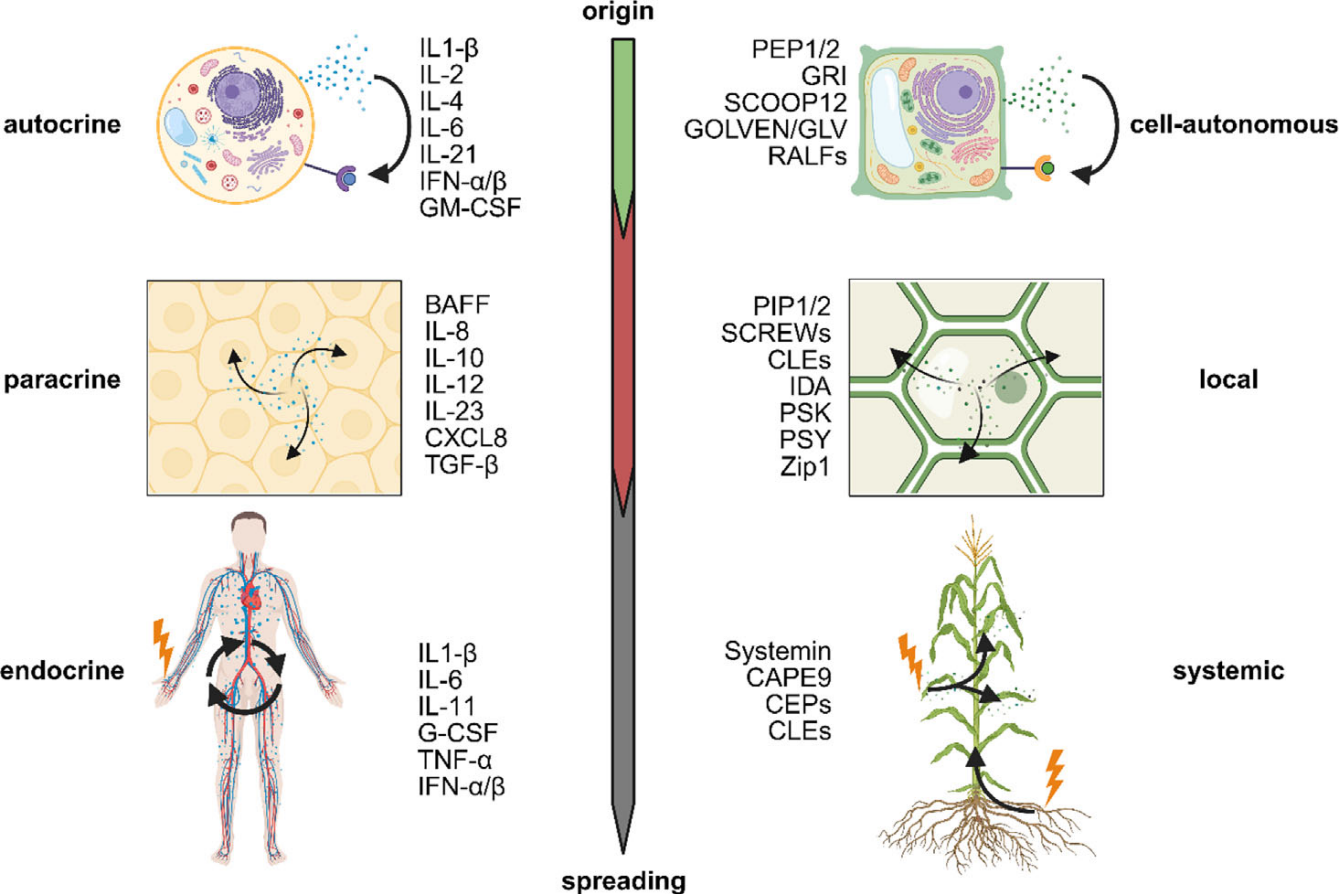
Spatial organization of peptide and cytokine signaling in plants and animals. Peptide signals are grouped according to their predominant spatial range of action, independent of receptor class or evolutionary origin. The central axis indicates increasing distance from the site of release. In plants, peptides are categorized as cell‐autonomous (acting at or near the site of origin), local (propagating within a tissue or organ without long‐distance vascular transport), or systemic (entering vascular tissues and mediating inter‐organ communication). Analogously, animal cytokines are organized into autocrine, paracrine, and endocrine categories based on their predominant physiological range. Asterisks indicate cytokines that can exhibit broader systemic activity under inflammatory conditions. This classification reflects predominant signaling behavior rather than strict boundaries, as spatial range may be context‐dependent. Figure was created with BioRender.

Cytokines bind to specific receptors mediating and regulating cell‐autonomous (autocrine), local (paracrine), and systemic (endocrine) responses (Figure 2). Cytokine receptors comprise transmembrane proteins with extracellular and cytoplasmic domains and include families such as type I/II, IL‐1, TNF, and chemokine receptors (Takeuchi, 2022). Phytocytokines are perceived at the cell surface by PRRs that belong mainly to two classes: receptor‐like kinases (RLKs) and receptor‐like proteins (RLPs), both of which have extracellular ligand‐binding domains anchored in the plasma membrane (e.g., LRR, LysM, lectin motifs) and initiate downstream signaling upon ligand recognition. RLKs transduce signals via an intracellular kinase domain, while RLPs lack this domain and signal through co‐receptors such as SOBIR1 and SERKs, analogous to multi‐subunit receptor systems in animals that recruit cytosolic kinases (Gust & Felix, 2014; van der Burgh et al., 2019). Plant RLKs and RLPs represent expansive receptor families with diverse extracellular domain structures, reflecting broad ligand recognition and functional specificity (reviews of plant surface receptors provide comprehensive background on the classification, mechanisms, and roles of RLKs and RLPs in immunity and development, for example, Ngou et al., 2024 and Tang et al., 2017).

Comparative analyses across 300 plant genomes classified RLKs into 18 families based on extracellular domain architecture (Yin et al., 2024), complementing specialized leucine‐rich repeat RLK (LRR‐RLK) resources. Most phytocytokine receptors identified to date belong to the RLK superfamily, predominantly the LRR‐RLK subclass that mediates perception of diverse endogenous peptides (Stegmann et al., 2017; Figure 1). Members of other RLK subfamilies, such as the CrRLK1L receptor FERONIA, perceive specific peptide ligands including RALFs, indicating that phytocytokine recognition is largely, though not exclusively, mediated by RLKs (Zhang, Yang, et al., 2020). Within the LRR‐RLK lineage, subclades such as LRR‐X and LRR‐XI perceive phytocytokines, whereas the closely related LRR‐XII subfamily was initially associated with exogenous MAMP recognition (e.g., FLS2 and EFR) (Greeff et al., 2012; Hosseini et al., 2020; Sampaio et al., 2025). However, recent studies show that members of the LRR‐XIIb subclade also detect endogenous danger peptides, demonstrating that self and non‐self‐cues are integrated within related receptor clades (Yu et al., 2025). Co‐evolution of phytocytokine signaling modules does not require symmetric sequence conservation between ligands and receptors. Receptors often reside in ancient LRR‐RK scaffolds that are conserved across species, whereas their peptide ligands exhibit greater sequence diversification (Bender & Zipfel, 2023; Man et al., 2023; Rhodes et al., 2022). Functional compatibility can nevertheless be maintained as long as key interaction determinants are preserved. Consistent with this, orthologous receptors such as CLV1/BAM, PSKR, and HAE/HSL2 remain conserved across angiosperms, while their corresponding peptide ligands frequently undergo duplication and motif diversification (Rhodes et al., 2022; Seo et al., 2024; Zhang et al., 2017). The SCOOP family illustrates how ligand diversification can generate distinct outputs through a shared receptor, analogous to chemokine systems in animals (Hou, Liu, Huang, et al., 2021; Rhodes et al., 2021; Wu et al., 2024).

Upon ligand perception, most phytocytokine receptors form heterodimeric complexes with SERK co‐receptors, such as BAK1/SERK3, to initiate downstream phosphorylation cascades (Gou & Li, 2020; Hohmann et al., 2018), with exceptions including PSYR1/2/3 (Ogawa‐Ohnishi et al., 2022; Zhang et al., 2024). Analogously, many mammalian cytokine receptors require accessory subunits, for example, IL‐6Rα/gp130 or the common γ‐chain shared by IL‐2, IL‐4, and IL‐7, to assemble functional signaling complexes (Boulanger et al., 2003; Leonard et al., 2019). Although plant and animal receptor systems evolved independently, both employ modular, co‐receptor‐dependent architectures to achieve signaling specificity.

In plants, one can distinguish three evolutionary tiers among phytocytokines. First, there are ancient and broadly conserved families such as CLE peptides (Bowman et al., 2017), C‐terminally encoded peptides (CEPs) (Taleski et al., 2021), RALFs (Campbell & Turner, 2017; Moussu et al., 2020) and SCREW peptides (Rhodes et al., 2022; Yamaguchi et al., 2010), with homologs documented across multiple angiosperm lineages, or even across land plants, including bryophytes (e.g., *Marchantia polymorpha*) and lycophytes (e.g., *Selaginella*). A second group are moderately conserved families, such as PSKs (Stührwohldt & Schaller, 2019a), PSY1‐type peptides (Amano et al., 2007), and IDA/IDL (Meng et al., 2020; Shi & Wang, 2018; Stø et al., 2015) which are largely restricted to vascular plants and show an angiosperm‐wide distribution, while being scarce or absent in bryophytes. Lastly, there are lineage‐specific or recently evolved families, including Peps, PIPs, CAPE peptides, Systemin, HypSys, and SCOOPs. While Peps and PIPs occur across many angiosperms and have expanded repertoires in Arabidopsis, maize, and rice, consistent with recent diversification in flowering plants, Systemin and HypSys are confined to *Solanaceae* and SCOOPs to *Brassicaceae* (Hou, Liu, Huang, et al., 2021; Lalun & Butenko, 2025; Lori et al., 2015; Ryan & Pearce, 2003; Stahl et al., 2022). Notably, Zip1 appears exclusive to maize, representing a species‐specific innovation (Depotter et al., 2022). Soybean encodes both conserved and lineage‐specific phytocytokines. Soybean harbors six AtPep1 orthologs (GmPep1‐GmPep6) that retain the conserved C‐terminal Pep motif and induce defense gene expression, ROS production, and extracellular alkalinization (Lee et al., 2018). This conservation indicates that the Pep‐PEPR danger‐signaling module predates *Fabaceae*‐*Brassicaceae* divergence (Lee et al., 2018). In addition, soybean has evolved lineage‐specific peptides such as GmSubPep, GmPep890, and GmPep914, which also activate defense responses, but are structurally distinct from GmPeps and are therefore presumed to signal through different, as yet unidentified receptors (Pearce et al., 2010; Yamaguchi et al., 2011). In poplar and willow, genes for antifungal phytocytokines and their receptors cluster in the genome, highlighting co‐evolution to coordinate immune responses against pathogens (Lintz et al., 2024). However, not all members of these peptide families function as phytocytokines. Many CLE peptides primarily regulate development, and some PIPs act outside immune signaling, indicating that phytocytokines represent functional subsets rather than entire peptide families (Creff et al., 2025; Toyokura et al., 2019). Across families, gene duplication and diversification, receptor co‐evolution, and stress adaptation act as major drivers. The phytocytokine landscape reflects both deep conservation of essential signaling modules (e.g., CLEs) and rapid, lineage‐specific diversification driven by pathogen pressures and developmental innovation (Lintz et al., 2024).

## PHYTOCYTOKINE IMMUNE MODULATION

Receptor–ligand binding activates signaling cascades, including co‐receptor complex formation, phosphorylation, MAPK activation, Ca^2+^ influx, and transcriptional changes. In the case of immune‐related phytocytokines these events upregulate defense genes, triggering canonical immune responses such as ROS production, cell wall reinforcement, and antimicrobial compound synthesis and often associate with phytohormonal signaling pathways (Hou, Liu, & He, 2021; Rzemieniewski & Stegmann, 2022; Figure 1). Similar to pro‐inflammatory and anti‐inflammatory cytokines, phytocytokines can modulate immunity inducing, amplifying or suppressing responses (Gust et al., 2017). Danger or immunity‐related phytocytokines are rapidly expressed after the perception of the required stimulus. For instance, Pep1, PIP1, SCOOP12, RGF7 and Zip1 induce the expression of their own precursor genes (Gully et al., 2019; Hou et al., 2014; Huffaker et al., 2006; Koenig, Sorger, Mantz, et al., 2025; Wang et al., 2021). Beyond autoregulation, some phytocytokines also induce other precursor genes, that is, PIP1 and Pep1 reciprocally activate each other's precursors (Hou et al., 2014), revealing a cross‐regulatory network. Phytocytokine signaling can fine‐tune receptor activity in different ways. For example, the Systemin‐related peptide anti‐Systemin acts as a competitive antagonist of the receptor SYR1, blocking co‐receptor recruitment and maintaining basal signaling balance (Cho et al., 2024; Wang, Maier, et al., 2025). Similarly, PSY peptides interact with the PSYR1/PSYR2/PSYR3 receptor module, where ligand binding suppresses the receptors' intrinsic basal activity rather than triggering activation (Ogawa‐Ohnishi et al., 2022). Conceptually, both modules illustrate how phytocytokines can modulate receptor signaling beyond canonical ligand‐induced activation, either by blocking active receptors or attenuating basal receptor activity, highlighting diverse strategies for maintaining homeostasis.

Although phytocytokines perform immune‐modulatory functions, they vary in their mechanisms of action. For example, Zip1, Peps, PIPs, SCOOPs, GRIp, and related peptides enhance immune responses by amplifying defense signaling and engaging classical pathways such as SA‐associated signaling and pattern‐triggered immunity (Hou, Liu, Huang, et al., 2021; Segonzac & Monaghan, 2019; Wrzaczek et al., 2015; Zelman & Berkowitz, 2023; Ziemann et al., 2018; Figure 1). In contrast, RALF peptides often act as negative regulators of immunity while promoting cell growth and cell wall dynamics (Bedinger et al., 2010; Liu, Liu, et al., 2024; Figure 1). Other phytocytokines, such as PSKs and PSYs, integrate developmental cues with environmental stimuli and stress responses (Liu, Jelenska, et al., 2024; Sauter, 2015). PSKs have been implicated in balancing growth and immunity in part by modulating transcriptional regulators and metabolic signaling, including the phosphorylation status of glutamine synthetase GS2, which contributes to uncoupling growth‐defense trade‐offs (Ding et al., 2023; Liu, Jelenska, et al., 2024). Through modulation of basal receptor activity, PSY perception by the PSYR module integrates growth promotion with stress‐responsive signaling pathways (Ogawa‐Ohnishi et al., 2022; Zhang et al., 2024). Similarly, CLE/CLEL peptides have roles beyond development, coordinating growth and immune processes; they can delay senescence, improve water and nutrient uptake, modulate interactions with beneficial symbiotic bacteria and fungi, and enhance resistance to various pathogens (Araya et al., 2014; Betsuyaku et al., 2011; Nakagami et al., 2024; Song et al., 2022).

Other phytocytokines, such as Systemin, HypSys, and CAPE1, are mostly involved in cell damage responses and are closely associated with JA‐mediated signaling (Chen et al., 2014; León et al., 2001; Ryan & Pearce, 2003; Segonzac & Monaghan, 2019; Figure 1). Recently, REF1 (REGENERATION FACTOR 1) was identified in tomato as a wound‐induced phytocytokine that links defense with a regenerative capacity (Yang et al., 2024). Unlike Peps and Systemin, which primarily modulate defense and wound‐associated signaling, the GRIM REAPER peptide (GRIP) stands out among characterized phytocytokines as the only peptide shown to directly induce programmed cell death (Wrzaczek et al., 2015). Treatment with GRIP causes visible lesions, elevated ion leakage, and activation of immune marker genes, indicating a strong link between peptide signaling and defense‐associated programmed cell death (Wrzaczek et al., 2015).

Recent work suggests that phytocytokines and microbe‐associated molecular patterns (MAMPs) trigger distinct immune responses, despite the activation of similar pathways (Koenig et al., 2023). Unlike microbial signals, phytocytokines do not induce cell death after tissue damage (Koenig et al., 2023; Zhou et al., 2020), although the underlying mechanisms remain unclear. A notable example is provided by SCREWs, which counteract MAMP‐ and ABA‐induced stomatal closure via the NUT/HSL3 receptor. By reopening stomata and promoting apoplastic water loss, SCREW signaling modulates the physiological context of pathogen colonization rather than simply reinforcing canonical MAMP‐triggered defenses (Liu et al., 2022). Future directions in the field should focus on dissecting how specificity of phytocytokine‐induced immune response is achieved at the mechanistic level, and how this is spatially orchestrated in a complex tissue. Resolving these questions will inform strategies to strengthen crop resistance against pathogens, pests, and abiotic stress, thereby improving agricultural productivity and sustainability.

### Mode of action of secreted peptide signals

Phytocytokines function as endogenous immune modulators that coordinate local and systemic defense responses. This tiered signaling allows precise spatial regulation of immunity, analogous to cytokine‐mediated communication in animals, while ensuring that defense responses are amplified only where needed (Hou, Liu, & He, 2021; Jian et al., 2024; Rhodes et al., 2021; Figure 2). Cytokine secretion in immune cells is highly regulated and cell type‐specific. For example, neutrophils rely mainly on *de novo* cytokine synthesis, whereas mast cells and eosinophils store pre‐formed cytokines for rapid release upon activation (Dong, 2021; Kaminska et al., 2024). Since plants lack specialized and mobile immune cells, it was therefore long unclear how peptide signaling could be spatially organized to support intercellular or long‐distance communication and how paracrine or endocrine‐like movement of peptide signals could be coordinated across tissues. Recent research using single‐cell multi‐omics analysis has identified different cell states in the context of plant–microbe interactions (Nobori et al., 2025). At immune‐active hotspots, for example, the center of an ETI region, a cell type named primary immune responder (PRIMER) shows non‐canonical immune signals whereas the bystander cells activate genes for long‐distance cell‐to‐cell immune signaling. PRIMER cells induce the transcription factor GT‐3A, which might be required for the proper induction of defense genes in surrounding cells. These two cell states propagate immune responses across the leaf (Nobori et al., 2025). In the context of phytocytokine signaling, we propose that PRIMER cells might perceive the stimuli, produce, and release phytocytokines, which are then perceived by bystander cells via cognate receptors thereby modulating immune responses (Figure 2). Building on the concept of local immune modulation, we classify phytocytokines according to their spatial propagation range, independent of receptor class or evolutionary origin. Cell‐autonomous peptides act primarily within the producing cell and, at most, its immediate neighboring cells, amplifying stress responses locally. Local peptides spread more broadly across a tissue or organ, coordinating responses among multiple cells, but do not enter the vasculature for long‐distance transport. Systemic peptides reach the vasculature, most often the phloem, and mediate inter‐organ communication. Some families, such as CLEs and RALFs, span multiple categories depending on expression domains and receptor distribution, forming a functional continuum rather than rigid distinctions. This functional classification helps frame how peptides operate at different spatial scales, from single cells to entire organs, and provides context for interpreting local versus systemic immune responses. In analogy to animals, cell‐autonomous peptides resemble autocrine signals, local peptides function similarly to paracrine signals, and systemic peptides parallel endocrine signaling, highlighting how plants can coordinate immune responses across tissues despite lacking specialized mobile immune cells (Figure 2). In this context, phytocytokines perceived by bystander cells function as medium‐range signals that induce local acquired resistance (LAR) in specific organs, such as the infected leaf. LAR is confined to the initial local infection site and does not spread throughout the organism, unlike systemic acquired resistance (SAR) (Ross, 1961).

SAR can be triggered by specific phytocytokines. For example, treatment with synthetic CAPE9 elevates SA levels and reduces bacterial pathogen infection locally. CAPE9 also acts systemically, traveling from the infection site to distant tissues to amplify the plant's “whole‐body” immune response, including stomatal immunity and resistance against *Pseudomonas syringae* (Chen et al., 2023). Systemin similarly functions systemically. Upon wounding or herbivore attack, Systemin moves through the phloem to distant tissues, where it regulates jasmonic acid biosynthesis and defense gene expression (Ryan & Pearce, 1998, 2003).

CEP peptides exemplify another endocrine‐like signaling mechanism. Produced in nitrogen‐deprived root zones, they are transported through the xylem to the shoot and perceived by phloem‐localized receptors CEPR1 and CEPR2. This triggers the production of secondary mobile peptides, such as CEPD1/2, which return to the roots to modulate nutrient uptake and growth (Ota et al., 2020; Rzemieniewski et al., 2024; Taleski et al., 2018, 2024). Pathogen‐induced CEP14 also enhances systemic disease resistance via CEPR2‐dependent signaling (Rzemieniewski et al., 2024; Wang, Yu, et al., 2025). Collectively, CEP peptides integrate developmental and immune cues through a vascular communication loop, acting as plant parallels to endocrine cytokine signaling in animals. Similarly, CLE peptides can act systemically to coordinate root‐shoot communication. For instance, root‐derived CLE peptides induced by rhizobial infection or mycorrhizal colonization travel via the xylem to the shoot, where they are perceived by receptor kinases (HAR1/SUNN/NARK) to suppress further nodulation (Nakagami et al., 2024; Yamaguchi et al., 2016). CLE mobility allows these peptides to function both as local developmental signals (e.g., stem cell maintenance in the SAM/RAM) and as long‐distance regulators of nodulation, phosphate homeostasis, and vascular patterning (Cornelis & Hazak, 2025; Nakagami et al., 2024; Okamoto et al., 2016).

### Phytocytokines in plant–microbe interactions

Plant pathogens have evolved diverse effector proteins that manipulate host immunity through multiple molecular mechanisms, extensively studied over the last two decades. Pathogens can manipulate the secretion of phytocytokines by inhibiting protease activity, producing their own proteases to degrade immune components or blocking signaling pathways (Misas‐Villamil et al., 2016). Microbial phytocytokine mimics (Table 2) can hijack host signaling pathways, promoting susceptibility or evading recognition (Hua et al., 2025; Petre et al., 2016; Toruño et al., 2016). For example, several *Fusarium* species and nematodes secrete RALF‐like peptides that structurally and functionally resemble plant RALF peptides and activate FERONIA‐dependent signaling to suppress host immunity and facilitate colonization (Masachis et al., 2016; Thynne et al., 2017; Wang, Liu, et al., 2024; Zhang, Peng, et al., 2020). Similarly, the arbuscular mycorrhizal fungus (AMF) *Rhizophagus irregularis* secretes the *Medicago truncatula* CLE16‐like peptide, RiCLE1, that attenuates ROS and promotes AMF colonization (Bashyal et al., 2025).

**Table 2:** Phytocytokine mimicry in plant–microbe interactions

Microbe (kingdom)	Microbial mimic	Plant peptide class	Host receptor/module	Mechanistic basis	Functional outcome	References
*Xanthomonas oryzae* pv. *oryzae* (Bacteria)	RaxX (Tyr‐sulfated)	PSY	XA21 RLK; PSY signaling module	Structural mimicry; Tyr‐sulfation required for activity	Growth modulation, virulence signaling	Pruitt et al. (2017)
*Fusarium oxysporum* (Fungi)	F‐RALF	RALF	FERONIA (CrRLK1L)	FER‐dependent, alkalinization	Immune suppression; enhanced disease	Wang, Liu, et al. (2024)
Cyst nematodes (Animalia)	CLE‐like	CLE	CLV/TDIF LRR‐RLKs	Processed to mature CLE mimics, receptor engagement	Feeding site formation; developmental reprogramming	Wang et al. (2011) and Guo et al. (2011)
*Rhizophagus irregularis* (Fungi)	CLE‐like	CLE	Host CLE signaling (MtCRN pathway)	CLE‐like suppresses ROS, enhances colonization	Promotes arbuscular mycorrhizal symbiosis	Bashyal et al. (2025)
Root‐knot nematode (Animalia)	IDL‐like (MiLDL1)	IDA	HAE/HSL2	Functional complementation of *ida* mutant	Cell separation; gall formation	Hu and Hewezi (2018)
*Rotylenchulus reniformis* (Animalia)	CEP‐like	CEP	CEPR1/2	CEP‐domain mimicry; nitrate signaling modulation	Root developmental reprogramming	Eves‐Van Den Akker et al. (2016)
*Fusarium* spp. (Fungi)	SCOOP‐like	SCOOP	MIK2‐BAK1/ SERK4	Conserved SxS motif; synthetic peptide activation	Immune activation or receptor hijacking	Rhodes et al. (2021)
*Ustilago maydis* (Fungi)	UmPR‐1La	CAPE	CAPE perception pathway	Host CathB3‐dependent cleavage releases CAPE‐like	Suppression of CAPE‐primed immunity	Lin et al. (2023)
Root‐knot nematode (Animalia)	PSY‐like	PSY	Similar to RaxX from *Xanthomonas*	Synthetic peptide bioactivity	Growth promotion	Yimer et al. (2023)
*Verticillium dahliae* (Fungi)	PSK‐like	PSK	PSKR1‐BAK1	Subtilase‐dependent PSK release	PTI suppression	Hua et al. (2025)

The *Verticillium dahliae* effector SCP8 promotes the production of the growth‐promoting phytocytokine PSK, likely by activating subtilases responsible for PSK release. PSK thereby enhances virulence through activation of the PSKR1‐BAK1 signaling complex suppressing PTI responses (Hua et al., 2025). An example of co‐evolution is maize smut *U. maydis* secreting UmPR‐1La, a PR1‐like protein with a C‐terminal domain resembling CAPE peptides, which engages host signaling to dampen defense while maintaining compatibility (Lin et al., 2023). Some microbial phytocytokine mimics perturb host peptide signaling, altering the timing, location, or intensity of defense responses in ways that promote pathogen colonization, rather than acting as classical agonists or antagonists. Similarly, mammalian pathogens deploy cytokine mimics to rewire host immunity. The helminth *Heligmosomoides polygyrus*, a naturally occurring intestinal roundworm, secretes Hp‐TGM, a functional TGF‐β mimic that binds mammalian TGF‐β receptors and drives regulatory T‐cell induction, dampening host inflammation (Johnston et al., 2017; White et al., 2021). Viruses such as herpes‐ and poxviruses encode virokines and viroceptors that act as decoys or subvert downstream signaling (Seet et al., 2003; Slobedman et al., 2009). These examples highlight that (phyto‐)cytokine mimicry can serve multiple purposes: (i) competitive inhibition of endogenous peptides at receptor sites, (ii) diversion of host signaling to manipulate host responses, and (iii) subversion of proteolytic processing or secretion routes critical for immune peptide activation.

As plants evolved complex repertoires of immune peptides and receptors, pathogens mirror this diversification by generating deceptive ligands. Most microbial phytocytokine mimics are biased toward developmental and growth‐related signaling peptides (CLEs, RALFs, PSYs, IDAs, CEPs), which microbes exploit to remodel host tissues and create infection niches. To date, only a few microbial peptides, such as the CAPE‐like peptides derived from the *U. maydis* UmPR‐1La and SCOOP‐like motifs identified in *Fusarium* proteomes, have been shown to interact with plant peptide perception systems in ways that affect immunity (Coleman et al., 2021; Lin et al., 2023). In *U. maydis*, CathB3‐mediated cleavage of UmPR‐1La releases CAPE‐like peptides that suppress plant defense and promote fungal virulence by subverting host CAPE‐primed immunity (Lin et al., 2023). SCOOP‐like sequences from *Fusarium* and certain bacteria can trigger MIK2‐dependent immune responses when tested as synthetic peptides, indicating that the MIK2 receptor perceives both endogenous SCOOP peptides and similar microbial motifs, although it is not yet established whether these motifs function as true virulence factors in natural infection (Rhodes et al., 2021). The SCOOP/MIK2 system thus highlights how conserved peptide motifs in microbes and plants can converge on the same receptor to activate defense signaling. These examples are likely the tip of the iceberg and future discoveries will likely reveal an underexplored dimension of phytocytokine mimicry. Unraveling the structural basis of phytocytokine mimics promises not only to shed light on plant–pathogen co‐evolution, but also to provide molecular tools for designing resistant crops by enhancing receptor specificity or engineering decoy receptors. Understanding the molecular basis of these mimics provides unique opportunities to uncover receptor specificities, refine our knowledge of peptide signaling networks, and exploit this information for engineering disease‐resistant crops (Boxes 1 and 2).

Box 1 **Bullet point summary**

- Cytokines and phytocytokines are small secreted peptides that mediate both local and systemic communication during immune and stress responses.
- They are synthesized as inactive precursors requiring proteolytic processing before receptor‐mediated signaling can occur.
- While animal cytokine maturation is well understood, phytocytokine processing, protease specificity, and cleavage‐site prediction remain major unresolved challenges.
- The mobility, long‐distance transport, and systemic signaling roles of phytocytokines are only beginning to be characterized.
- Comparative analyses reveal convergent evolutionary solutions in immunogenic peptide signals, highlighting key knowledge gaps in our understanding of how plants process, release, transport, and perceive these immune peptides.

Box 2 **Open questions**

1. What molecular features determine the specificity of phytocytokine signaling?
2. How are multiple peptide signals integrated to coordinate spatiotemporal immune responses across different cell types?
3. Do plant‐associated microbes perceive phytocytokines, and if so, how does this affect host–microbe interactions?
4. How can machine‐learning models be designed to predict phytocytokine‐processing proteases despite overlapping and low‐specificity cleavage motifs?
5. Are phytocytokines sensed exclusively by surface receptors, or do alternative perception mechanisms exist?

## CONFLICT OF INTEREST

The authors declare no conflicts of interest.

## Data Availability

Data sharing not applicable to this article as no datasets were generated or analysed during the current study.
